# CSMed^®^ wound dressing for prophylaxis and management of radiation dermatitis in breast and head–neck cancer patients: a single hospital prospective clinical trial

**DOI:** 10.1007/s00432-024-05624-6

**Published:** 2024-02-23

**Authors:** Yueh-Chun Lee, Hsien-Chun Tseng, Huei-Fang Yang, Yi-Hung Lee, Ya-Fang Ko, Shin-Tsung Chang, Hsin-Lin Chen, Bo-Jiun Chang, Ying-Hsiang Chou

**Affiliations:** 1 Department of Radiation Oncology, Chung Shan Medical University Hospital, No. 110, Sec. 1, Jianguo N. Rd., South Dist., Taichung, 40201 Taiwan; 2 School of Medicine, Chung Shan Medical University, Taichung, 40201 Taiwan; 3 Department of Nursing, Chung Shan Medical University Hospital, Taichung, 40201 Taiwan; 4 Institute of Medicine, Chung Shan Medical University, Taichung, 40201 Taiwan; 5 Department of Medical Imaging and Radiological Sciences, Chung Shan Medical University, Taichung, 40201 Taiwan

**Keywords:** CSMed^®^, Breast cancer, Head-and-neck cancer, Radiation dermatitis, Radiotherapy

## Abstract

**Purpose:**

CSMed^®^ wound dressing, a dressing with various herb extracts, was tested for its therapeutic effect in radiation dermatitis of breast and head-and-neck cancer patients.

**Methods:**

This study included 20 breast cancer patients and 10 head-and-neck cancer patients. Half of the irradiated area was covered with CSMed^®^ and the other half was under routine treatment. The severity of radiation dermatitis was evaluated with radiation therapy oncology group (RTOG) grade throughout the treatment and the follow-up period. The RTOG grade between the dressed and undressed area were compared to illustrate the therapeutic effect of CSMed^®^ dressing.

**Results:**

The results showed that CSMed^®^ dressed area had significant lower RTOG score at 3–7 weeks and final record during the treatment, and 1–3 weeks during follow-up than undressed area.

**Conclusions:**

This indicated that CSMed^®^ can delay the onset, reduce the severity, and enhance healing of radiation dermatitis. CSMed^®^ can be used for prophylaxis and management of radiation dermatitis.

**Supplementary Information:**

The online version contains supplementary material available at 10.1007/s00432-024-05624-6.

## Introduction

Radiation dermatitis is the most common side effect after radiotherapy. Patients with radiation therapy usually receive multiple small doses of radiation to kill cancer cell while minimizing damage to surrounding healthy cells. Depending on the radiation dosage and the tissue sensitivity of patients, radiation dermatitis manifests within days or weeks after radiation exposure. Radiation dermatitis is restricted to the exposure area and the skin changes at the boundary are demarcated sharply (Leventhal and Young 2017). Although there is still no standard procedure in preventing and treating radiation dermatitis, patients are advised to avoid cosmetic products, sun exposure, wearing tight-fitting clothing, and extreme heat or cold(Hegedus et al. 2017). These advices do prevent the condition of radiation dermatitis from deterioration clinically, but the damaged tissues still need to recover without any support.

Various methods can be used for prevention and management of radiation dermatitis. Among these methods, using dressing on irradiated skin can maintain a moist environment and promote healing and re-epithelialization of the wound (McQuestion 2011). In addition, different therapeutic ingredients can be added onto the dressing, which can further promote wound healing and ease the uncomfortability.

Addressing the challenges in treating radiation dermatitis, our team has developed CSMed^®^ wound dressing. This innovative dressing, composed of bio-cellulose, herbal extracts, and γ-PGA, offers notable anti-inflammatory and wound healing properties. In this study, we evaluate its efficacy in treating patients with breast or head-and-neck cancer, aiming to provide clinical evidence for its effectiveness and insights for potential future improvements.

## Methods

Patients arranged to receive radiation therapy between August 2020 and December 2020 were recruited for this study. The inclusion criteria were (1) patient were diagnosed with breast cancer or head-and-neck cancer; (2) patients aged between 20 and 70; and (3) patients underwent radiation therapy. The exclusion criteria were (1) patients had dermatitis or burns which is not induced by radiation therapy; (2) patients whom did not apply CSMed^®^ dressing for 2 consecutive days, necessary to ensure consistent treatment efficacy; (3) patients whom cannot complete the whole treatment and follow-up procedures; and (4) patient who are allergic to the ingratiation of the CSMed^®^ dressing. A written informed consent with respect to the use of topical agents and clinical data management for research purposes was obtained from all participants. This trial was designed and conducted in accordance with the principles outlined in the Declaration of Helsinki and within the guidelines of Good Clinical Practices (trial registration numbers: NCT06001463, August 23, 2023). This trial was approved by the Institutional Review Board of the Chung Shan Medical University Hospital (212250-017-F-001).

### Study design

The size and position of breast or head-and-neck cancer were stimulated and target contoured via computed tomography. The physicians determined the dosage and treatment time in intervals rather than a single generic dose based on the results. Radiologists conducted the radiation therapy followed a fractionation regimen and patients received weekly irradiation for 4–8 weeks in treatment and were followed-up for another 4 weeks. The radiation dosage was recorded and the irradiated area was labeled. An irradiated area of 11 cm*14 cm with best fit or easy accessibility was chosen for CSMed^®^ wound dressing application, suited to typical radiation areas in head and neck and breast cancer treatments. The area without dressing was treated with routine skin care in each patient. This routine care involved daily cleansing of the skin with warm water, along with instructions for patients to avoid other dressings, cosmetic products, direct sun exposure, tight-fitting clothing, and exposure to extreme temperatures. The CSMed^®^ dressing application started from the first day of treatment and till the day of the first follow-up. The dressing was renewed daily. The patients were also asked to avoid other dressing, cosmetic products, sun exposure, wearing tight-fitting clothing, and extreme heat or cold. The skin condition between dressed and undressed area were compared for each patient.

### Composition and properties of CSMed^®^

CSMed^®^ wound dressing is made from bio-cellulose membrane and herbal extracts of *Aloe vera*, *Centella asiatica*, and *Abelmoschus esculentus*. Purified γ-PGA is also included in the ingredients. Thus, CSMed^®^ wound dressing has the ability of anti-inflammation, antioxidation, angiogenesis, and wound healing.

### Patient evaluation

The severity of acute radiation dermatitis was graded using the radiation therapy oncology group (RTOG) clinical grading standard. Grade 0 indicates no visible change to the skin. Grade 1, characterized by faint erythema or dry desquamation, was assigned a score of 1 in this study. Grade 2a includes bright erythema or dry desquamation, along with sore, itchy, and tight skin, and was assigned a score of 2. Grade 2b, featuring patchy moist desquamation, yellow/pale green exudate, soreness, and oedema, was assigned a score of 3. Grade 3, which involves moist desquamation other than in skin folds and creases, was assigned a score of 4. Finally, Grade 4, characterized by skin necrosis or ulceration of full thickness dermis, was assigned a score of 5. The RTOG scores were assessed weekly by radiation oncologists. For a detailed view of this scoring system, please refer to Supplemental Table 1.

### Statistical analysis

Categorical data are presented as *n* (%) and performed Fisher’s exact test. Continuous data are presented as the median (range) and analyzed by the Wilcoxon signed rank test. Furthermore, we assessed the association of patients’ characteristics with RTOG level using generalized estimating equations (GEE) with a first-order auto-regression covariance matrix. Data management and statistical analyses were conducted using SAS version 9.4 software (SAS Institute, Inc.).

## Results

A total of 30 patients were included in this study, and 20 patients were diagnosed with breast cancer and the other 10 patients were diagnosed with head-and-neck cancer. Table 1 shows the demographic characteristics of the patients. All of the breast cancer patients were women (*n* = 20), and all but one head-and-neck cancer patients were male (*n* = 9). Most of the patients with head-and-neck cancer live with their spouses.

**Table 1:** Patients’ characteristics in baseline

Variable	ALL (*n* = 30)		Breast cancer (*n* = 20)		Head and neck cancer (*n* = 10)		*p* value
	*n*	(%)	*n*	(%)	*n*	(%)	
Gender							< 0.001
Male	9	(30)	0	(0)	9	(90)	
Female	21	(70)	20	(100)	1	(10)	
Age (years)							0.160
31–40	4	(13.33)	3	(15)	1	(10)	
41–50	13	(43.33)	11	(55)	2	(20)	
51–60	8	(26.67)	3	(15)	5	(50)	
61–70	5	(16.67)	3	(15)	2	(20)	
BMI (kg/m^2^)							0.875
BMI < 18.5	0	(0)	0	(0)	0	(0)	
18.5 ≤ BMI < 24	14	(47)	10	(50)	4	(40)	
24 ≤ BMI < 27	8	(27)	5	(25)	3	(30)	
27 ≤ BMI < 35	8	(27)	5	(25)	3	(30)	
BMI ≥ 35	0	(0)	0	(0)	0	(0)	
Marital status							0.137
Unmarried	6	(20)	1	(10)	5	(25)	
Married	20	(67)	9	(90)	11	(55)	
Divorced	4	(13)	0	(0)	4	(20)	
Cohabitating	0	(0)	0	(0)	0	(0)	
Widowed	0	(0)	0	(0)	0	(0)	
Living status							**0.007**
Solitary	3	(10)	3	(15)	0	(0)	
Live with spouse	13	(43)	4	(20)	9	(90)	
Live with child	4	(13)	3	(15)	1	(10)	
Live with spouse and child	6	(20)	6	(30)	0	(0)	
Others	4	(13)	4	(20)	0	(0)	
Educational level							0.400
Illiterate	0	(0)	0	(0)	0	(0)	
Junior high school and below	5	(17)	3	(15)	2	(20)	
Senior high school	16	(53)	9	(45)	7	(70)	
College	8	(27)	7	(35)	1	(10)	
Graduate and above	1	(3)	1	(5)	0	(0)	
Occupation							0.062
Public officials	2	(6.67)	2	(10)	0	(0)	
Construction	1	(3.33)	0	(0)	1	(10)	
Service industry	6	(20)	6	(30)	0	(0)	
Manufacturing	3	(10)	2	(10)	1	(10)	
Freelance	3	(10)	2	(10)	1	(10)	
Housekeeper	4	(13.33)	3	(15)	1	(10)	
Health care worker	1	(3.33)	1	(5)	0	(0)	
Business	1	(3.33)	1	(5)	0	(0)	
Unemployed	3	(10)	1	(5)	2	(20)	
Retirement	4	(13.33)	0	(0)	4	(40)	
Others	2	(6.67)	2	(10)	0	(0)	

Significant values are showing in bold

The comparison of the score converted from RTOG grade in skin with dressed and undressed area during the treatment period and the follow-up period are shown in Table 2. At the beginning of the treatment period, there was no difference in RTOG grade due to all patients have a zero score. The score converted from RTOG grade in skin with dressing was significantly lower than undressed skin at 3–7 weeks and the final record (all *p* < 0.05) during the treatment periods. During the follow-up periods, similar results were found at 1–3 weeks (all *p* < 0.001). For all patients, the median cumulative RT dose was 60.4 Gy (range 55–70.4 Gy) and the median cumulative RT frequency was 28.5 times (range 25–35 times) during the treatment periods.

**Table 2:** Score converted from radiation therapy oncology group (RTOG) grade between dressing and undressing area

	Dressing		Undressing		
	*N*	Score	*N*	Score	*p* Value
Treatment period (week)					
1	30	–	30	–	NS^a^
2	30	–	30	–	NS^a^
3	30	0 (0–0)	30	0 (0–1)	**0.031**
4	30	0 (0–1)	30	1 (0–2)	** < 0.001**
5	28	1 (0–2)	28	1 (0–3)	** < 0.001**
6	20	1 (0–3)	20	1.5 (1–4)	** < 0.001**
7	11	2 (1–4)	11	2 (1–5)	**0.016**
8	4	4 (2–4)	4	4 (3–5)	0.500
Final	30	1 (0–4)	30	2 (1–5)	** < 0.001**
Cumulative RT dose	30	55 (46.8–70.4)			
Cumulative RT frequency	30	28.5 (20–35)			
Follow period (week)					
1	30	1 (0–3)	30	2 (1–4)	** < 0.001**
2	30	0 (0–2)	30	1 (0–3)	** < 0.001**
3	30	0 (0–1)	30	1 (0–2)	** < 0.001**
4	30	0 (0–1)	30	0 (0–2)	0.063
Cumulative RT dose	30	60.4 (55–70.4)			
Cumulative RT frequency	30	32.5 (25–35)			
Final cumulative RT dose	30	60.4 (55–70.4)			
Final cumulative RT frequency	30	32.5 (25–35)			

Data are presented as median (range) and performed by Wilcoxon signed rank test. Significant values are showing in bold

^a^RTOG grade was all 0

The subgroup analyses by cancer types are shown in Table 3. For breast cancer patients, the score converted from RTOG grade in skin with dressing was significantly lower than undressed area at 3–6 weeks and the final record (all *p* < 0.05) during the treatment periods. For head-and-neck cancer patients, the score converted from RTOG grade in skin with dressing was significantly lower than undressing at 4–7 weeks (all *p* < 0.05). For the two types of cancer patients, similar results were found at 1–3 weeks (all *p* < 0.05) during the following periods.

**Table 3:** Score converted from radiation therapy oncology group (RTOG) grade between dressing and undressing by cancers

	Breast cancer					Head and neck cancer				
	Dressing		Undressing			Dressing		Undressing		
	*N*	Score	*N*	Score	*p* value	*N*	Score	*N*	Score	*p* value
Treatment period (week)										
3	20	0 (0–0)	20	0 (0–1)	**0.031**	10	0 (0–0)	10	0 (0–0)	NS
4	20	0 (0–1)	20	1 (0–2)	**0.001**	10	0 (0–0)	10	1 (0–1)	**0.031**
5	18	1 (0–2)	18	1.5 (1–3)	** < 0.001**	10	0 (0–2)	10	1 (0–3)	**0.004**
6	10	0.5 (0–1)	10	1 (1–2)	**0.008**	10	1 (0–3)	10	2 (1–4)	**0.016**
7	1	1 (1–1)	1	2 (2–2)	1.000	10	2 (1–4)	10	2.5 (1–5)	**0.031**
8	0	–	0	–	–	4	4 (2–4)	4	4 (3–5)	0.500
Final	20	1 (0–4)	20	2 (1–5)	** < 0.001**	10	2 (1–4)	10	3 (1–5)	0.125
Cumulative RT dose	20	54 (46.8–60)				10	66 (60–70.4)			
Cumulative RT frequency	20	25 (20–30)				10	33 (30–35)			
Follow period (week)										
1	20	1 (0–3)	20	2 (1–4)	** < 0.001**	10	1 (0–3)	10	2 (1–4)	**0.008**
2	20	0 (0–2)	20	1 (0–3)	** < 0.001**	10	0 (0–2)	10	1 (0–3)	**0.004**
3	20	0 (0–1)	20	1 (0–2)	** < 0.001**	10	0 (0–1)	10	1 (0–2)	**0.031**
4	20	0 (0–1)	20	0 (0–2)	0.250	10	0 (0–0)	10	0 (0–1)	0.500
Cumulative RT dose	20	55 (55–60.4)				10	66 (66–70.4)			
Cumulative RT frequency	20	25 (25–33)				10	33 (32–35.0)			
Final cumulative RT dose		55 (55–60.4)				10	66 (66–70.4)			
Final cumulative RT frequency		25 (25–33)				10	33 (32–35)			

Data are presented as median (range) and performed by Wilcoxon signed rank test. Significant values are showing in bold

We further analyzed the relationship between patients’ characteristics and the risk of RTOG level (Table 4). The score greater than 0 was defined as a worst event. GEE analysis revealed that patients in week 4 and week 5 had significantly higher odds ratio (OR) (6.12- or 29.47-fold, respectively) for worse events than in week 3. Compared with undressing, skin with dressing significantly reduced the OR for the worse event (OR = 0.20, 95% CI 0.15–0.27). Further multivariate analysis revealed that the skin with dressing still significantly reduced the OR for the worse event (OR = 0.07, 95% CI 0.03–0.18).

**Table 4:** Relationship between Score converted from radiation therapy oncology group (RTOG) grade and patient and cancer characteristics

Variable	Score 1–5 vs. Score 0			
	Univariate		Multivariate	
	OR (95% CI)	*p* value	OR (95% CI)	*p* value
Gender				
Male	1		1	
Female	1.84 (0.97–3.49)	0.064	2.97 (0.95–9.31)	0.062
Cancer type				
Breast	1			
Head and neck	0.61 (0.33–1.13)	0.114		
Treatment period (week)				
3	1		1	
4	**6.12 (2.78–13.47)**	** < 0.001**	**8.01 (3.65–17.58)**	** < 0.001**
5	**29.47 (11.37–76.4)**	** < 0.001**	**69.49 (14.89–324.31)**	** < 0.001**
Cumulative RT dose	0.96 (0.92–1.01)	0.147		
Dressing				
No	1		1	
Yes	**0.20 (0.15–0.27)**	** < 0.001**	**0.07 (0.03–0.18)**	** < 0.001**

Performed by generalized estimating equation (GEE) with a first-order auto-regression covariance matrix. Significant values are showing in bold

Adjusted for gender due to the biological importance of the variables in multivariate analysis

## Discussion

In this prospective study, CSMed^®^ wound dressing had proved to be an excellent dressing for radiation dermatitis treatment. Our results indicated that CSMed^®^ delayed the onset of radiation dermatitis for more than 1 week and area covered with CSMed^®^ showed significant lower score than undressed area during weeks 3–7 of treatment. During the follow-up period, CSMed^®^ also led to significant lower RTOG grade and shorter healing time. In short, irradiated area covered with CSMed^®^ developed milder syndrome of radiation dermatitis and healed quicker than untreated area.

Compared with other studies which used double-blind or compared the effectiveness among different subject groups, this study was conducted and compared on the same patient. Since half of the radiated area was covered with CSMed^®^ dressing, and the other half was not dressed and treated with routine procedure, the difference of personal sensitivity and response against radiation or treatment were eliminated by this procedure. The therapeutic results can then be compared directly between dressed and undressed area.

The radiation dosage in the conventional breast cancer therapy is 45–50 Gy (Kim and Algan 2021) and the dosage can be increased to 60 Gy in standard dose fractionation regimen (Chakraborty and Chatterjee 2021). The dosage for intensity modulated radiation therapy of head-and-neck cancer is between 50 and 60 Gy (Murthy et al. 2017). In this study, the average radiation dosage for breast cancer and head-and-neck cancer were 54 Gy (ranged between 46.8 and 60) and 66 Gy (ranged between 60 and 70.4), respectively. Comparing these data, the dosage in this study was higher than others. This indicates that CSMed^®^ can have an outstanding effect in a high dosage regimen, and CSMed^®^ can surely have the same effect in a routine radiation therapy.

The supportive material of a dressing can be made by wool, cotton, collagen, cellulose and its derivatives, plastics, rubber, and composite materials (Chattopadhyay and Raines 2014; Sharma et al. 2014). Among those materials, several strains of *K. xylinus* produce extracellular cellulous forming a biofilm of varying thickness to serves as a protective barrier against drying, natural enemies and radiation (Portela et al. 2019). This bio-cellulous has advantages in purity, biocompatibility, biodegradability, non-toxicity, higher surface area, elasticity, resistance, and flexibility (Klemm et al. 2001; Rahman and Netravali 2016). And it has the capacity to retain moisture, absorb exudates from the injured tissue and accelerate granulation (Khalid et al. 2017; Li et al. 2015).

Medical plants are used for treating diseases and wounds for thousands of years. Bioactive constituents in medicinal plants are responsible for the therapeutic activities, and these phytochemicals have unlimited potential for application (Asha and Thomas 2019). They can be taken orally directly. They can also be extracted and added onto dressings for wound healing and tissue regeneration. *Aloe vera* is a medicinal plant traditionally used in many countries for disease and skin lesion treatment (Hekmatpou et al. 2019; Shelton 1991). Aloe vera contains chemicals which can improve wound healing and cytokine production. Aloe vera can also inhibit inflammation, itching, and irritation (Bunyapraphatsara et al. 1996; Heck et al. 1981; Somboonwong et al. 2000). *Centella asiatica* is known for its nutritional and therapeutic properties (Arribas-López et al. 2022). Due to having antimicrobial, antioxidant, anti-inflammatory, neuroprotective, and wound healing properties (Prakash et al. 2017; Tan et al. 2021). It is used for the treatment of dermatoses and skin lesions by improving collagen synthesis (Brinkhaus et al. 2000; Maquart et al. 1999) and microcirculatory function (Cesarone et al. 2001; Incandela et al. 2001). *Abelmoschus esculentus* is an herbal medicine used to treat wound healing with antioxidant, antibacterial, and anti-inflammation properties (Nesa et al. 2014). This is due to the phenolic compounds like saponin, tannin, alkaloid, and flavonoid in the fruit (Sipahi et al. 2022).

Previous studies showed that ultra-high molecular weight poly-γ-Glutamic Acid (γ-PGA) successfully stimulated corneal wound healing by inducing an inflammatory effect, enhancing cell migration and cell proliferation(Bae et al. 2010). A wound healing study in rat showed γ-PGA also promoted production of transforming growth factor-beta (TGF-β), keratinocyte growth factor (KGF), and β-catenin, collagen cross-linking, angiogenesis, and re-epithelialization, and formation of hair follicles (Choi et al. 2015).

Despite the good results, there are several limitations in this study. First of all, the sample size was small. Only 30 patients were included in this study. Second, only breast cancer and head-and-neck cancer patients were included in the study. Radiation therapy can be conducted on other cancer patients, and radiation dermatitis may occur in other part of skin. In the future, we will recruit more patients and make sure the findings in this study remain valid. In addition, different treatment strategies may be applied to check the curative effect. For example, applying the dressing in an earlier stage of treatment, such as one week before radiation therapy. It is also reasonable to examine the effectiveness of this dressing on other cancer patients.

## Conclusion

The effectiveness of CSMed^®^ wound dressing on reducing the severity and increasing the healing of radiation dermatitis in patients with breast cancer and head and neck cancer shows its potential in prophylactic application.

## Supplementary Information

Below is the link to the electronic supplementary material.

Supplementary file1 (DOCX 13 KB)

## Data Availability

All data generated during this study are included in this published article.

## References

[CR1] Arribas-López E, Zand N, Ojo O, Snowden MJ, Kochhar T (2022) A systematic review of the effect of *Centella asiatica* on wound healing. Int J Environ Res Public Health 19(6):3266 35328954 10.3390/ijerph19063266 PMC8956065

[CR2] Asha KK, Thomas S (2019) Recent advances on herb-derived constituents-incorporated wound-dressing materials: a review. Polym Adv Technol 4:823–839

[CR3] Bae SR, Park C, Choi JC, Poo H, Kim CJ, Sung MH (2010) Effects of ultra high molecular weight poly-gamma-glutamic acid from *Bacillus subtilis* (chungkookjang) on corneal wound healing. J Microbiol Biotechnol 20(4):803–808 20467257

[CR4] Brinkhaus B, Lindner M, Schuppan D, Hahn EG (2000) Chemical, pharmacological and clinical profile of the East Asian medical plant *Centella asiatica*. Phytomedicine 7(5):427–448 11081995 10.1016/s0944-7113(00)80065-3

[CR5] Bunyapraphatsara N, Jirakulchaiwong S, Thirawarapan S, Manonukul J (1996) The efficacy of Aloe vera cream in the treatment of first, second and third degree burns in mice. Phytomedicine 2(3):247–251 23194624 10.1016/S0944-7113(96)80050-X

[CR6] Cesarone MR, Belcaro G, De Sanctis MT, Incandela L, Cacchio M, Bavera P, Ippolito E, Bucci M, Griffin M, Geroulakos G et al (2001) Effects of the total triterpenic fraction of *Centella asiatica* in venous hypertensive microangiopathy: a prospective, placebo-controlled, randomized trial. Angiology 52(Suppl 2):S15-18 11666117

[CR7] Chakraborty S, Chatterjee S (2021) Adjuvant radiation therapy in breast cancer: recent advances & Indian data. Indian J Med Res 154(2):189–198 35295008 10.4103/ijmr.IJMR_565_20 PMC9131773

[CR8] Chattopadhyay S, Raines RT (2014) Review collagen-based biomaterials for wound healing. Biopolymers 101(8):821–833 24633807 10.1002/bip.22486 PMC4203321

[CR9] Choi JC, Uyama H, Lee CH, Sung MH (2015) Promotion effects of ultra-high molecular weight poly-γ-glutamic acid on wound healing. J Microbiol Biotechnol 25(6):941–945 25791849 10.4014/jmb.1412.12083

[CR10] Heck E, Head M, Nowak D, Helm P, Baxter C (1981) Aloe vera (gel) cream as a topical treatment for outpatient burns. Burns 7(4):291–294

[CR11] Hegedus F, Mathew LM, Schwartz RA (2017) Radiation dermatitis: an overview. Int J Dermatol 56(9):909–914 27496623 10.1111/ijd.13371

[CR12] Hekmatpou D, Mehrabi F, Rahzani K, Aminiyan A (2019) The effect of aloe vera clinical trials on prevention and healing of skin wound: a systematic review. Iran J Med Sci 44(1):1–9 30666070 PMC6330525

[CR13] Incandela L, Cesarone MR, Cacchio M, De Sanctis MT, Santavenere C, D’Auro MG, Bucci M, Belcaro G (2001) Total triterpenic fraction of Centella asiatica in chronic venous insufficiency and in high-perfusion microangiopathy. Angiology 52(Suppl 2):S9-13 11666128

[CR14] Khalid A, Ullah H, Ul-Islam M, Khan R, Khan S, Ahmad F (2017) Bacterial cellulose-TiO_2_ nanocomposites promote healing and tissue regeneration in burn mice model. RSC Adv 7:47662–47668

[CR15] Kim CS, Algan O (2021) Radiation therapy for early stage breast cancer. StatPearls Publishing, Treasure Island, FL 29083774

[CR16] Klemm D, Schumann D, Udhardt U, Marsch S (2001) Bacterial synthesized cellulose—artificial blood vessels for microsurgery. Prog Polym Sci 26:1561–1603

[CR17] Leventhal J, Young MR (2017) Radiation dermatitis: recognition, prevention, and management. Oncology (Williston Park) 31(12): 885–887, 894–889 29297172

[CR18] Li Y, Jiang H, Zheng W, Gong N, Chen L, Jiang X, Yang G (2015) Bacterial cellulose-hyaluronan nanocomposite biomaterials as wound dressings for severe skin injury repair. J Mater Chem B 3(17):3498–3507 32262233 10.1039/c4tb01819b

[CR19] Maquart FX, Chastang F, Simeon A, Birembaut P, Gillery P, Wegrowski Y (1999) Triterpenes from *Centella asiatica* stimulate extracellular matrix accumulation in rat experimental wounds. Eur J Dermatol 9(4):289–296 10356407

[CR20] McQuestion M (2011) Evidence-based skin care management in radiation therapy: clinical update. Semin Oncol Nurs 27(2):e1-17 21514477 10.1016/j.soncn.2011.02.009

[CR21] Murthy V, Gurram L, Kannan S, Gandhi M, Gupta T, Laskar SG, Budrukkar A, Agarwal JP (2017) Elective nodal dose of 60 Gy or 50 Gy in head and neck cancers: a matched pair analysis of outcomes and toxicity. Adv Radiat Oncol 2(3):339–345 29114601 10.1016/j.adro.2017.06.005 PMC5605312

[CR22] Nesa M, Islam MM, Alam MB (2014) Analgesic, anti-inflammatory and CNS depressant activities of the methanolic extract of *Abelmoschus esculentus* Linn. seed in mice. Br J Pharm Res 4(7):849–860

[CR23] Portela R, Leal CR, Almeida PL, Sobral RG (2019) Bacterial cellulose: a versatile biopolymer for wound dressing applications. Microb Biotechnol 12(4):586–610 30838788 10.1111/1751-7915.13392 PMC6559198

[CR24] Prakash V, Jaiswal N, Srivastava M (2017) A review on medicinal properties of *Centella asiatica*. Asian J Pharm Clin Res 10(10):69

[CR25] Rahman MM, Netravali AN (2016) Aligned bacterial cellulose arrays as “green” nanofibers for composite materials. ACS Macro Lett 5(9):1070–1074 35614647 10.1021/acsmacrolett.6b00621

[CR26] Sharma S, Dua A, Malik A (2014) Third generation materials for wound dressings. Int J Pharm Sci Res 5(6):2113–2124

[CR27] Shelton RM (1991) Aloe vera. Its chemical and therapeutic properties. Int J Dermatol 30(10):679–683 1823544 10.1111/j.1365-4362.1991.tb02607.x

[CR28] Sipahi H, Orak D, Reis R, Yalman K, Şenol O, Palabiyik-Yücelik SS, Deniz İ, Algül D, Guzelmeric E, Çelep ME et al (2022) A comprehensive study to evaluate the wound healing potential of okra (*Abelmoschus esculentus*) fruit. J Ethnopharmacol 287:114843 34801610 10.1016/j.jep.2021.114843

[CR29] Somboonwong J, Thanamittramanee S, Jariyapongskul A, Patumraj S (2000) Therapeutic effects of Aloe vera on cutaneous microcirculation and wound healing in second degree burn model in rats. J Med Assoc Thai 83(4):417–425 10808702

[CR30] Tan SC, Bhattamisra SK, Chellappan DK, Candasamy M (2021) Actions and therapeutic potential of Madecassoside and other major constituents of *Centella** asiatica*: a review. Appl Sci 11:8475

