# Supplementary material for: CSMed® wound dressing for prophylaxis and management of radiation dermatitis in breast and head–neck cancer patients: a single hospital prospective clinical trial
Source: J Cancer Res Clin Oncol. 2024 Feb 23;150(2):101. doi: 10.1007/s00432-024-05624-6 (PMC10891181; doi:10.1007/s00432-024-05624-6)
Supplement: Supplementary file 1 — Supplementary file1 (DOCX 13 KB) [file 432_2024_5624_MOESM1_ESM.docx]

| **Grade** | **Observation** | **Score** |
| --- | --- | --- |
| 0 | No visible change to the skin | 0 |
| 1 | Faint erythema or dry desquamation | 1 |
| 2a | Bright erythema/dry desquamation. Itchy and tight. | 2 |
| 2b | Patchy moist desquamation. Yellow/pale green exudate. Soreness and oedema. | 3 |
| 3 | Moist desquamation other than in skin folds and creases. | 4 |
| 4 | Skin necrosis or ulceration of full thickness dermis. | 5 |

Supplemental Table 1. RTOG Scoring Criteria.
